# Dynamics of Small Non-coding RNA Profiles and the Intestinal Microbiome of High and Low Weight Chickens

**DOI:** 10.3389/fmicb.2022.916280

**Published:** 2022-06-30

**Authors:** Hao Zhou, Lingyu Yang, Jinmei Ding, Ke Xu, Jiajia Liu, Wenqi Zhu, Jianshen Zhu, Chuan He, Chengxiao Han, Chao Qin, Huaixi Luo, Kangchun Chen, Yuming Zheng, Christa F. Honaker, Yan Zhang, Paul B. Siegel, He Meng

**Affiliations:** ^1^Shanghai Collaborative Innovation Center of Agri-Seeds, School of Agriculture and Biology, Shanghai Jiao Tong University, Shanghai, China; ^2^Department of Animal and Poultry Sciences, Virginia Tech, Blacksburg, VA, United States; ^3^Carilion Clinic, Roanoke, VA, United States

**Keywords:** small non-coding RNA, intestinal bacteria, co-evolution, host-microbiota interactions, cecum, chicken

## Abstract

The host and its symbiotic bacteria form a biological entity, holobiont, in which they share a dynamic connection characterized by symbiosis, co-metabolism, and coevolution. However, how these collaborative relationships were maintained over evolutionary time remains unclear. In this research, the small non-coding RNA (sncRNA) profiles of cecum and their bacteria contents were measured from lines of chickens that have undergone long-term selection for high (HWS) or low (LWS) 56-day body weight. The results from these lines that originated from a common founder population and maintained under the same husbandry showed an association between host intestinal sncRNA expression profile (miRNA, lncRNA fragment, mRNA fragment, snoRNA, and snRNA) and intestinal microbiota. Correlation analyses suggested that some central miRNAs and mRNA fragments had interactions with the abundance of intestinal microbial species and microbiota functions. miR-6622-3p, a significantly differentially expressed (DE) miRNA was correlated with a body weight gain related bacterium, *Alistipes putredini*s. Our results showed that host sncRNAs may be mediators of interaction between the host and its intestinal microbiome. This provides additional clue for holobiont concepts.

## Introduction

The host and its symbiotic intestinal microbes share complex relationships in which they interact with each other and affect traits associated with growth, development, and health of the host (McFall-Ngai et al., 2013; Selosse et al., 2014; Foster et al., 2017). Together, they form one distinct biological entity (Rosenberg and Zilber-Rosenberg, 2014, 2018), defined as a “holobiont.” In recent years, this concept has earned considerable support in a variety of studies (Fraune and Bosch, 2010; Gilbert et al., 2012; Bordenstein and Theis, 2015). In a holobiont, there are complex symbiotic interactions, co-metabolism, and coevolution between a host and its microbiome (Rosenberg and Zilber-Rosenberg, 2014, 2018; Zhou et al., 2022). However, the way hosts and microbes engage in cooperative dialog to maintain these reciprocal interactions during development and evolution remains unknown.

Small non-coding RNAs (sncRNAs) in eukaryotes are a large family of endogenously expressed transcripts, 18–200 nucleotides long, that play an important role in regulating cell function. They include miRNA, transfer RNA (tRNA), small nucleolar RNA (snoRNA), small nuclear RNA (snRNA), and piwi protein-associated RNA (piRNA), lncRNA fragment, and mRNA fragment, which are considerably more diverse than those found in prokaryotes (Bloch et al., 2017). Among all classes of sncRNAs, miRNAs are the most studied. Emerging findings have shown that miRNAs play important roles in communication of the host and its intestinal microbiome (Liu et al., 2016). They stated that fecal miRNAs secreted by the intestine may enter intestinal bacteria and thereby control bacterial gene expression and growth. Fecal miRNAs can also be used as markers for microbial fluctuations along with intestinal pathology in the intestine (Moloney et al., 2018). Moreover, the bi-directional host-microbiome interaction was mediated by miRNAs in colorectal cancer (Yuan et al., 2018). These findings imply potential roles that sncRNAs may have in mediating the host and intestinal microbe’s interactions to preserve their coevolution and co-metabolism relationships.

Here, we used the Virginia body weight chicken lines as a model to investigate mechanisms behind host-microbiota communication during evolution. The lines were divergently selected for a single trait, 56-day body weight, for 56 generations, resulting in an approximately 15-fold difference between high weight selected (HWS) and low weight selected (LWS) lines (Siegel, 1962; Dunnington and Siegel, 1996; Márquez et al., 2010; Zhou et al., 2022). We combined molecular data of small non-coding RNA sequencing and metagenomic sequencing based on this model. Questions addressed were: (1) Will the expression of intestinal sncRNAs and the abundance of intestinal microbiota adaptably change during long-term artificial selection? (2) Is it possible that different kinds of intestinal sncRNAs mediate in interactions between the host, and its intestinal microbiota during evolution?

## Materials and Methods

### Animals and Sample Collections

Protocols used for this experiment were approved by the Institutional Animal Care and Use Committee at Virginia Tech. The chickens used in this experiment were from generation 56 of the Virginia HWS and LWS lines (Siegel, 1962; Dunnington and Siegel, 1996; Márquez et al., 2010). These lines, which originated from a common founder population of White Plymouth Rock chickens, have been subjected to divergent selection for either high or low 56-day body weight. Husbandry was consistent through all generations. Chicks from each line were penned individually in the same building. They were moved to individual cages with gently sloping wire floors at 19 weeks of age. They were fed starter (0–8 weeks), development (8–19 weeks), and breeder (thereafter) antibiotic-free corn-soybean mash diets. At generation 56, The 56-day body weights (mean ± SD) were 1,848 ± 160 g and 130 ± 23 g for HWS and LWS males, respectively. The 56-day body weights (mean ± SD) for females were 1,510 ± 160 g and 92 ± 26 g. Cecal tissues and their content samples were collected from 10 HWS (5 males and 5 females) and 10 LWS (5 males and 5 females) chosen at random at 245 days of age. All cecal tissues were harvested and put into liquid nitrogen. The cecal contents were temporarily stored at 4°C before DNA extraction.

### Metagenomic Sequencing

Microbial genomic DNAs were isolated from the cecal contents following a previously reported protocol (Li et al., 2017). Metagenomic DNA paired-end libraries were prepared with an insert size of 350 base pairs (bp). Sequencing was performed on Illumina HiSeq 2500 platform.

### Metagenomic Data Analyses

Metagenome assembly and construction of the raw reads were cleaned by Kneaddata to exclude adapter sequences, low-quality sequences, and contaminated DNA, including host genomic DNA^1^. The average error rate of the clean reads was less than 0.001. Short reads (length <75 bp) and unpaired reads were also excluded to form clean reads. For each sample, the clean reads were assembled by Megahit (v1.0.6) under pair-end mode (Li et al., 2016), and gene prediction was performed on contigs larger than 500 bp by Prodigal (v2.6.3)^2^ with the parameter “–metagenome –kingdom Bacteria,” and gene models with CDS length less than 102 bp were filtered out. Then, assembly and gene prediction were performed on the 20 samples individually, using the same methods for each sample. A non-redundant gene catalog was constructed using the gene models predicted from each sample and each group by cd-hit-est (v4.8.1) (Li and Godzik, 2006). Finally, we obtained a total of 2,227,868 non-redundant genes.

To calculate relative gene abundance, the clean reads from each sample were aligned against the gene catalog by salmon (v0.14.1) (Patro et al., 2017), with the default parameters. Sequence-based abundance profiling was performed as previously described (Qin et al., 2012). Carbohydrate-active Enzyme (CAzy), Clusters of Orthologous Groups (COG), Enzyme code (EC), Gene Ontology (GO), Kyoto Encyclopedia of Genes and Genomes ortholog database (KEGG ko), and KEGG pathway analyses were carried out by emapper (v2.0.0) (Huerta-Cepas et al., 2016). Their relative abundances were calculated by summing the abundance of the respective genes belonging to each category per sample, based on each annotation, respectively.

To identify prokaryotic species and estimate their relative abundance, we used the Metagenomic Phylogenetic Analysis (MetaPhlAn) toolbox (v2.0) (Segata et al., 2012) to provide a picture of the complex bacteria and archaea community. Next, alpha diversities of microbial community were measured using species richness and abundance-based coverage estimator (ACE) index. The overall differences in the bacterial community structures were evaluated by non-metric multidimensional scaling (NMDS) based on Bray-Curtis dissimilarity values and performed with the “Phyloseq” (v1.30.0) (McMurdie and Holmes, 2013) package in R.

### Small Non-coding RNA Sequencing

Total RNA from cecal tissues was extracted using TRIzol reagent (Invitrogen Life Technologies, Carlsbad, CA, United States) according to the manufacturer’s protocol. The RNA integrity was determined using a 2100 Bioanalyzer (Agilent Technologies, Santa Clara, CA, United States).

Small non-coding RNA sequencing libraries were prepared directly from the validated cecal total RNA using TruSeq Small RNA Library Preparation Kit as per the manufacturer’s instruction (Illumina, San Diego, CA, United States). Samples were sequenced with the Illumina HiSeq 2000 platform using the 1 × 50 bp single-end read method of Illumina sequencing.

### Small Non-coding RNA Data Analysis

After sequencing, raw data were obtained from the 20 samples. We initially cut the adapter off the raw sequencing data and trimmed the low-quality bases of each sequence as the clean data using Trimmomatic (Bolger et al., 2014). The following options were used for trimming: MAXINFO 15:0.8, MINLEN 15. SncRNA data analysis was carried out by Unitas, using software default parameters (Gebert et al., 2017), to classify and annotate chicken miRNA (known miRNA annotated in chicken), other species miRNA (known miRNA annotated in other species), piRNA, rRNA, tRNA, mRNA fragments, lncRNA fragments, snoRNA, snRNA, miscellaneous RNA (miscRNA), low complex RNA, and non-annotated RNA.

Then, the abundance of each sncRNA annotated was calculated using transcript per million reads [RPM = (the number of reads that can be matched to each RNA)/(the number of total RNA reads) × 10^6^]. We utilized the DEseq2 R package (Love et al., 2014) to perform sncRNA differential expression analysis on HWS vs. LWS. The *p*-value < 0.05 was considered as significantly differentially expressed (DE).

To investigate the function of the DE chicken miRNAs of HWS and LWS, we performed the analysis of gene target prediction for DE chicken miRNAs using miranda (v3.3a) with the parameters of “-en –20 -strict.” The 3′UTR sequence of the protein coding genes in chicken GRCg6a genome (GCA_000002315.5)^3^ was used as the input target dataset. KEGG pathway and GO enrichment analysis of target genes were implemented by DAVID using human database (v6.8)^4^. For both GO and KEGG pathway, a *p*-value < 0.05 was considered as statistically significant.

### Weighted Gene Co-expression Analysis of Small Non-coding RNAs

Weighted gene co-expression analysis (WGCNA, v1.68) (Langfelder and Horvath, 2008) was performed under the subgroup-specific signatures to identify potential sncRNA modules or hub sncRNAs associated with body weight. Each type of sncRNA (see above) was analyzed separately but was collectively referred to as sncRNAs. The soft thresholding power was set as 5, 24, 24, and 10 for chicken miRNA, other species miRNA, mRNA fragments, and snoRNA, respectively, to ensure scales-free *R*^2^ = 0.85 for correlations. The snRNA and lncRNA fragments whose soft thresholding power did not fit the criteria of *R*^2^ = 0.85 were excluded for the next analysis. Then the miRNA, other species miRNA, mRNA fragments, and snoRNA were clustered hierarchically and classified into modules based on their measured network distance, known as topological overlap. Module-trait associations were estimated using the correlation between the module eigengene and the phenotype (body weight), which allows easy identification of expression set (module) highly correlated to the phenotype. Modules whose module eigengene (ME) exhibited the highest positive or negative correlation with body weight were selected as candidate modules to be studied. In this study, hub genes, highly interconnected with nodes in a module, were defined by module connectivity, measured by the absolute value of the Pearson’s correlation (cor.geneModuleMembership >0.8) and clinical trait relationship, measured by the absolute value of the Pearson’s correlation (cor.geneTraitSignificance >0.2). We identified hub genes in the module that were highly correlated with body weight.

### Correlation Analyses of Hub Small Non-coding RNAs and Microbiome

We performed correlation analyses using Spearman’s correlation for bacteria at the species level and the hub sncRNAs as well as for microbial involved pathways and hub sncRNAs, respectively. R functions of cor() and corr.test() were used to calculate Spearman’s correlation coefficient and corresponding *p*-value, respectively. Significance was defined as a correlation coefficient (r) of over | 0.5| with a *p*-value of 0.05 for species-sncRNA correlations, and r of over | 0.6| with a *p*-value of 0.01 for microbial pathway-sncRNA correlations. The network constructed for them is visualized with Cytoscape (v3.6.1) (Shannon et al., 2003).

## Results

### Composition of Intestinal Microbiota of High Weight Selected and Low Weight Selected

**FIGURE 1:**
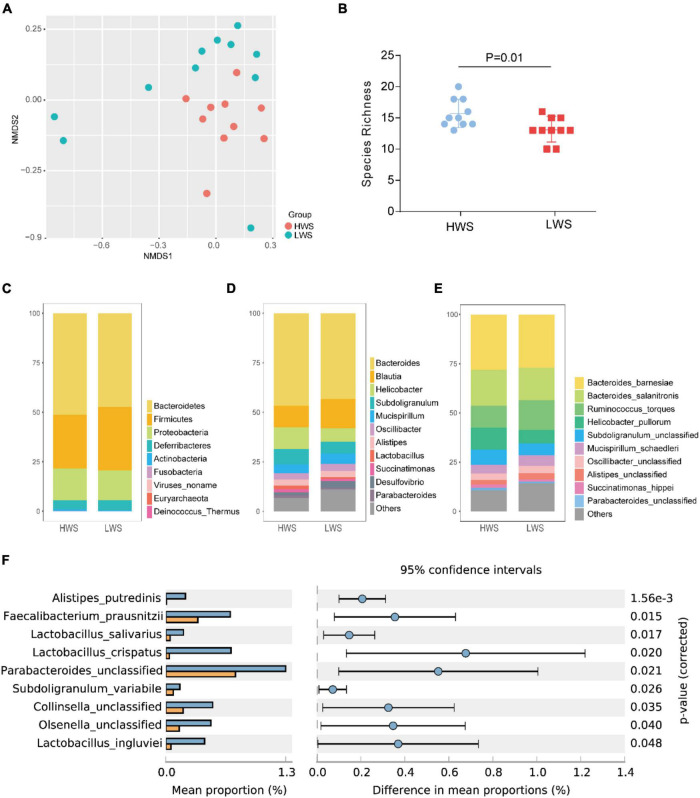
The composition and structure of intestinal microbiota in HWS and LWS. **(A)** The NMDS plot of intestine bacterial profiles. **(B)** Intestinal bacteria species richness. **(C)** Composition of intestinal microbiota at the phyla level, **(D)** genus level, and **(E)** species level. Some low abundant intestinal bacteria were classified as “others” in **(D,E)**, detailed information of these intestinal bacteria was shown in Supplementary Table 2E **(D)** and Supplementary Table 2F **(E)**. **(F)** Comparisons of intestinal microbiota abundance at the species level in HWS and LWS. Blue and orange bars are HWS and LWS, respectively.

Metagenomic data were used to calculate the Beta and Alpha diversity of the intestinal microbiota in each line. After sequencing, the raw metagenomic data underwent a series of preprocessing steps before analysis, which is described in Supplementary Material. The Beta diversity analysis of microbial communities in HWS and LWS showed clear dissimilarities between them (Figure 1A). Alpha diversity, the microbial diversity in each sample, was evaluated based on species richness and ACE index. Species richness (Student’s *t*-test, *p* = 0.01, Figure 1B) was higher in HWS than LWS. Also, the ACE index revealed significantly greater diversity for the community of HWS than LWS (Student’s *t*-test, *p* = 0.019). These observations demonstrate more microbial diversity in HWS than LWS.

### Taxonomic Changes in High Weight Selected and Low Weight Selected Microbiomes

To further investigate the intestinal microbe community features of HWS and LWS, we carried out the taxonomic assignment for the metagenomic data using MetaPhlAn2. The relative abundance of each taxon between the two groups were compared. In total, 25 bacterial taxa were differentially abundant between the groups, including 1 phyla, 1 class, 1 order, 2 families, 4 genera, 9 species, and 4 strains (Welch’s test, *p* < 0.05, Supplementary Table 2). At the phyla level, the bacterial composition was dominated by Bacteroidetes (51% in HWS and 47% in LWS) and Firmicutes (27% in HWS and 32% in LWS), followed by Proteobacteria (16% in HWS and 13% in LWS) (Figure 1C and Supplementary Table 2A). Only Actinobacteria were significantly different between the two lines, being enriched in HWS. At the genus level, the bacterial composition was dominated by *Bacteroides* in both lines (47% in HWS and 43% in LWS) (Figure 1D and Supplementary Table 2E). All the significantly different abundant genera, *Olsenella, Parabacteroides*, and *Faecalibacterium*, were enriched in HWS. The two most abundant species were *Bacteroides barnesiae* (28% in HWS and 27% in LWS) and *Bacteroides salanitronis* (18% in HWS and 17% in LWS), both from the *Bacteroides* genus (Figure 1E and Supplementary Table 2F). Of the 9 species that were significantly different in abundance between HWS and LWS, none was significantly increased in abundance in LWS (Figure 1F and Supplementary Table 2F). Of the species that increased significantly in abundance in HWS, *Alistipes putredinis* was most abundant in HWS (*p* = 0.0016). *Lactobacillus crispatus*, *Lactobacillus ingluviei*, and *Lactobacillus salivarius* belong to genera of *Lactobacillus*, suggesting that *Lactobacillus* plays an important role in HWS intestines. Taken together, these data show that intestinal microbiota changed in chickens selected long-term for high or low body weight.

### Differences in Small Non-coding RNAs Profiles in High Weight Selected and Low Weight Selected Intestines

**FIGURE 2:**
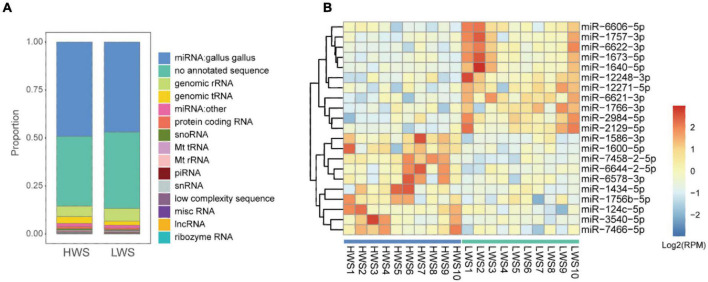
**(A)** The bar chart of the proportion of total abundance of different types of sncRNAs in HWS and LWS. **(B)** Heatmap of the expression level of 25 significantly differentially expressed chicken miRNAs (|fold change| > 2 and adjusted *p*-value < 0.05) in HWS and LWS (*n* = 10). The color in the heatmap represents the log 2 of expression values (RPM). The orange color represents a higher expression value than average expression across samples, while the blue color represents the opposite.

The 20 raw data sets of sncRNAs generated by deep sequencing are detailed in Supplementary Table 1A. Eleven types of sncRNAs were expressed in intestines of HWS and LWS (Figure 2A and Supplementary Table 4). Chicken miRNAs accounted for approximately half of the abundance of small RNAs (49% in HWS and 47% in LWS). A large proportion of reads failed to map any database (36% in HWS and 40% in LWS). Genomic rRNA were the third most enriched sncRNAs and accounted for 6% in both lines.

To identify specific sncRNAs differentially expressed between the two lines, five common studied sncRNAs were investigated. We detected 1,417 chicken miRNAs, 15,808 other species miRNAs, 1,635 lncRNA fragments, 10,111 mRNA fragments, 200 snoRNAs, and 40 snRNAs (Supplementary Table 5). After the comparison, we found that all of these types of sncRNAs in the intestine seem to be affected by the long-term divergent selection for body weight. There were 208 chicken miRNAs, 762 other species miRNAs, 1,189 mRNA fragments, 32 lncRNA fragments, 17 snoRNAs, and 5 snRNAs differentially expressed between HWS and LWS (*p*-value < 0.05, Supplementary Table 6). Of the 208 DE chicken miRNAs, 126 and 82 were upregulated and downregulated in HWS ceca, respectively, compared to LWS (Supplementary Table 6C). Among them, 21 reliable DE chicken miRNAs were filtered with | fold change| > 2 and adjusted *p*-value < 0.05 (Figure 2B). The most significantly upregulated miRNAs in the intestine of LWS were miR-1673-5p and miR-1640-5p, while miR-7458-2-5p and miR-3540-5p were most significantly upregulated in the intestine of HWS (*p* < 0.005, Figure 2B). In addition, for the comparison in mRNA fragments, 694 downregulated DE mRNAs fragments and 495 upregulated DE mRNA fragments were identified in HWS (*p* < 0.05, Supplementary Table 6D). The detailed results of differential expression analysis for other sncRNAs are presented in Supplementary Table 6.

### Functional Analysis of Differentially Expressed Small Non-coding RNAs

We predicted the target genes of 21 reliable DE chicken miRNAs between HWS and LWS on the chicken genome (| fold change| > 2 and adjusted *p*-value < 0.05) and found 3,518 targeted chicken genes. Further GO analysis showed that there were 7,242 GO terms significantly enriched with target genes, of which 392 GO terms were significantly related, including terms related to biological processes, cellular components, and molecular functions. Biological process analyses showed that the large groupings of target genes were significantly related to functions, such as regulation of lipid metabolism (GO:0019216), response to nutrients (GO:0007584), response to nutrient levels (GO:0031667), digestive tract development (GO:0048565), and digestive system development (GO:0055123). Similarly, the KEGG pathways of the target genes are shown in Supplementary Table 7B. Fifty-one pathways were significantly enriched, of which including several pathways involved in cecal functions of digestion, absorption, and metabolism, including biosynthesis of unsaturated fatty acids, glycolysis/gluconeogenesis, fatty acid metabolism, and fat digestion and absorption. These four pathways involve 19 genes, *PGK1, ACSS2, HK1, PLA2G1B, DGAT2, PKM, PGAM1, FASN, LDHA, PLA2G2A, PLA2G12B, ACOX1, ALDH1A3, SCD, ACSL5, PFKL, SCD5, ACOT7*, and *HACD2*.

Consistently, functional analysis of 1,189 DE mRNA fragments revealed that 202 GO functions (173 biological processes, 13 cellular components, 16 molecular functions) and 9 KEGG pathways were significantly enriched (Supplementary Table 7C). Biological Process (BP) GO terms, including lipid digestion, negative regulation of muscle hypertrophy, and adipose tissue development, were observed. Similar results were obtained in the KEGG pathway assigned, including protein digestion and absorption, fat digestion and absorption, and carbohydrate digestion and absorption. These findings suggest that the miRNAs and mRNA fragments altered in the intestines of HWS and LWS may regulate target genes to influence cecal function.

### Co-expression Analyses of Small Non-coding RNAs

**FIGURE 3:**
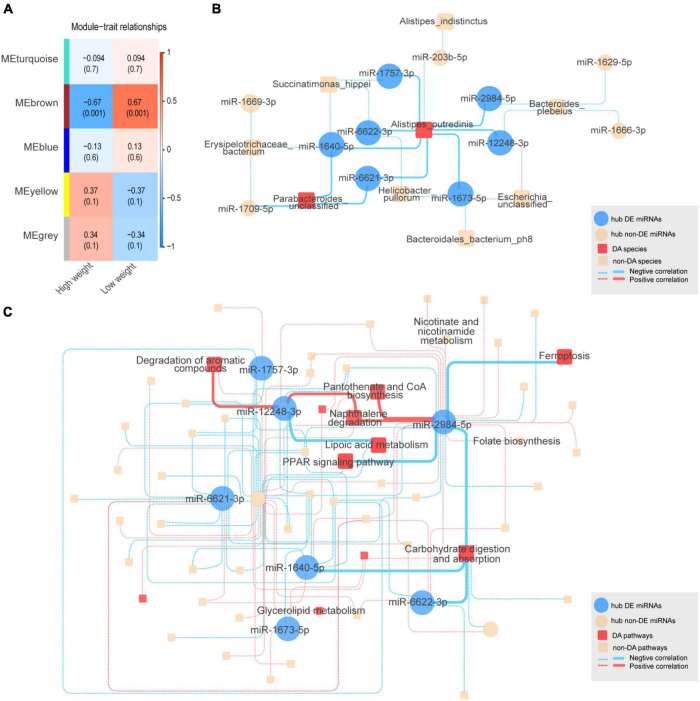
**(A)** Chicken miRNA co-expression module and body weight correlations with corresponding *p* values (in parentheses). The left panel shows five modules with different colors. The color scale on the right shows module-trait correlation from –1 (blue) to 1 (red). **(B)** The complex interactive relationships between hub chicken miRNAs and intestinal microbiota. Blue solid circles represent significantly differentially expressed (DE) hub miRNAs between HWS and LWS. Yellow solid circles represent non-DE hub miRNAs. Red squares represent significantly differentially abundant (DA) microbial species. Red and blue lines represent the respective positive and negative correlations between miRNAs and microbial species. The interactions between DE hub miRNAs and DA species are represented by solid lines, with other interactions shown as dashed lines. **(C)** KEGG pathway. Blue solid circles represent significantly differentially expressed (DE) hub miRNAs between HWS and LWS. Yellow solid circles represent non-DE hub miRNAs. Red squares represent significantly differentially abundant (DA) pathways. Red and blue lines, respectively, represent the positive and negative correlations between miRNAs and microbial pathways. The interactions between DE hub miRNAs and DA pathways are shown as solid lines, with other interactions by dashed lines.

To identify co-expressed sncRNA modules that are important for phenotypic variation of body weight in HWS and LWS, clusters of co-abundant sncRNAs were identified using the R package WGCNA (Langfelder and Horvath, 2008). The modules closely related to high and low body weight were of particular interest. Each type of sncRNA (chicken miRNAs, miRNAs annotated in other species, lncRNA fragments, mRNA fragments, snoRNAs, and snRNAs) was analyzed separately. The number of co-expressed modules and modules significantly related to body weight are shown in Table 1. Only miRNA and mRNA fragment profiles were significantly related to body weight (*p* < 0.05). Of the modules identified in the chicken miRNA profiles (Figure 3A), miRNA profiles in other species, and mRNA fragment profiles, the 1, 2, and 1 modules were significantly associated with body weight, respectively (*p* < 0.05, Table 1). Subsequently, we set out to identify the central nodes (hub chicken miRNAs) in the significantly associated chicken miRNA co-expression module, MEbrown module, (Figure 3A) by selecting chicken miRNAs with the highest module membership scores. These miRNAs could have important roles in the ceca of HWS. The 12 hub miRNAs identified in the MEbrown module were miR-6622-3p, miR-1669-3p, miR-1666-3p, miR-203b-5p, miR-1640-5p, miR-1709-5p, miR-1757-3p, miR-1673-5p, miR-12248-3p, miR-6621-3p, miR-2984-5p, and miR-1629-5p (Supplementary Table 8A). In addition, 36 annotated in other species, and 20 mRNA fragments were identified as hub sncRNAs (Supplementary Table 8).

**TABLE 1:** Summary of the quantitative information obtained from 5 types of sncRNA analyses.

Type of sncRNA	Expressions^a^	DEs^b^	Modules^c^	Significant modules^d^	Hub genes^e^
miRNA (chicken)	1,417	208	5	1	12
miRNA (other species)	15,808	762	16	2	36
mRNA fragments	1,635	1,189	6	1	20
lncRNA fragments	10,111	17	0	0	0
snoRNA	200	5	2	0	0
snRNA	40	32	0	0	0

*Expressions^a^, sncRNAs expressed.*

*DEs^b^, Significantly differentially expressed sncRNAs (p < 0.05).*

*Modules^c^, Co-expressed modules identified.*

*Significant modules^d^, Co-expressed modules identified (p < 0.05).*

*Hub genes^e^, Hub miRNAs in co-expressed modules identified.*

### Correlation Analyses of Hub Small Non-coding RNAs With the Intestinal Microbiome

To investigate the relationships between hub sncRNAs and the intestinal microbiome, 12 hub miRNAs and 75 microbial species were analyzed together. Of the 27 edges generated between them (| *r*| < 0.5, *p* < 0.05) (Figure 3B and Supplementary Table 9A), miR-6622-3p, a DE miRNA with lower expression levels in HWS, was negatively correlated with *Alistipes putredinis*, which was the species with higher abundance in HWS than LWS. Additionally, *Alistipes putredinis* was negatively correlated with 8 miRNAs. Seven of them (miR-6622-3p, miR-6621-3p, miR-2984-5p, miR-1757-3p, miR-1673-5p, miR-1640-5p, miR-12248-3p), had significantly downregulated expression in HWS (Supplementary Tables 6C, 9A). A total of 351 pathways were used to correlate the functional composition of the microbiome data with 12 hub miRNAs (| *r*| < 0.6, *p* < 0.06). Eleven hub miRNAs were significantly correlated with 57 pathways (Figure 3C and Supplementary Table 9B). Carbohydrate digestion and absorption, a pathway significantly enriched in HWS, was negatively correlated with 5 hub miRNAs (Supplementary Table 9B). Among them, miR-1640-5p, miR-2984-5p, and miR-6622-3p had lower expression in HWS than LWS. miR-12248-3p was downregulated in HWS and connected to lipoic acid metabolism that had high abundance in HWS. Based on these findings, miRNAs may selectively affect the growth of certain bacteria. Thus, bacteria correlated with DE miRNAs are likely to be related to body weight of HWS and LWS chickens.

We then investigated correlations of microbial species and pathways with hub mRNA fragments (Supplementary Tables 9C,D). Similar to the results of miRNAs, several hub mRNA fragments were significantly correlated with differentially enriched microbial species in HWS and LWS. These included *Alistipes putredinis, Lactobacillus salivarius*, and *Lactobacillus ingluviei*, which were highly enriched in HWS (Supplementary Table 9C). In the analysis of mRNA fragments correlated with microbial pathways (Supplementary Table 9D) pyruvate metabolism, lipoic acid metabolism, and carbon metabolism were more abundant in HWS than LWS. They were negatively correlated with five genes, *CPA5, CPA1, CTRL, CTRC*, and *AMY2A*, which were downregulated in HWS.

## Discussion

The Virginia body weight lines of chickens provide an ideal model to investigate the holobiont, a distinct biological entity formed by the host and its symbiotic microbes, as well as their complex relationships (Rosenberg and Zilber-Rosenberg, 2014, 2018). These chickens were raised under the same conditions and originated from a common founder population (Siegel, 1962). The difference is that HWS and LWS chickens have developed different genotypes under the pressure of long-term artificial selection, and their body weight in the generation studied (S56) differed by 15-fold. The genetics underlying the response to selection within these lines are well documented and demonstrate that many loci contribute to the observable differences in body weight (Wahlberg et al., 2009; Sheng et al., 2015; Lillie et al., 2018). Lillie et al. (2018) applied genome resequencing to several generations from the HWS and LWS and found 14.2% of the genome showed extreme differentiation between them, within 395 genomic regions. Host genetics have been reported to influence the structure of intestinal microbiome communities, which in turn affects host metabolism (Goodrich et al., 2014). We also found that the abundance of intestinal microbiota was significantly altered between HWS and LWS. For example, *Alistipes putredinis*, *Lactobacillus crispatus*, *Lactobacillus ingluviei*, and *Lactobacillus salivarius* were highly enriched in the intestine of HWS compared to LWS. In humans, a significantly higher level of Lactobacillus species was also observed in obese than lean patients (Armougom et al., 2009). *Alistipes putredinis* is a producer of short-chain fatty acids (SCFAs) and can be associated with body weight in the chickens, piglets, rabbits, and calves (Du et al., 2018; Yu et al., 2019; Fang et al., 2020; Wang et al., 2020). In addition, the pathways related to energy metabolism involved with intestinal microbiota were different in HWS and LWS, including carbon metabolism, carbon fixation pathways in prokaryotes, pyruvate metabolism, fatty acid degradation, and carbohydrate digestion and absorption. These findings were consistent with our previous study that artificial selection can also induce adaptive changes in intestinal microbiota (Zhao et al., 2013; Meng et al., 2014), and most of the changed intestinal microbes were associated with body weight (Zhao et al., 2013). Taken together, it appears that the host and its intestinal microbes are closely related during evolution, and they form a holobiont that adapts to its encountered selection.

Small non-coding RNAs are a key mode of molecular communication that have an important role in interactions and symbiosis of the host and its intestinal microbiome (Liu et al., 2016; Zhao et al., 2017; Johnston et al., 2018). To investigate the roles of sncRNAs between the host and its intestinal symbiotic microorganisms throughout the evolution process, we first examined the sncRNA profiles expressed in the intestines of HWS and LWS and found major changes in the expression of all five types of sncRNAs between the two lines. Further co-expression analyses of these sncRNA forms revealed the presence of co-expressed modules of 3 miRNA and 1 mRNA fragment modules substantially related to body weight. These findings suggest that the sncRNA profiles, especially in miRNAs and mRNA fragments expressed in the intestine, could be affected by long-term artificial selection for body weight. One of the hub miRNAs in the module associated with body weight, miR-203b, has been reported to be growth-related in fish (Yan et al., 2013; Tu et al., 2017). It could inhibit muscle development in tilapia (Yan et al., 2013) and impede cell growth in Chinese perch (Tu et al., 2017) by controlling body weight-related gene expression. In our study, miR-203b was significantly downregulated in HWS, suggesting that it may have a role in the growth of chickens. The single nucleotide polymorphism of precursor of another hub miRNA, miR-1757, was associated with semi-evisceration weight, evisceration weight, carcass weight, and body weight in chickens (Li et al., 2015). Functional analysis of DE miRNAs also revealed that they were involved in regulating the metabolism-related functions and pathways such as response to nutrient levels, digestive tract development, and digestive system development, suggesting their crucial roles in body weight. Unlike miRNA, the functions of mRNA fragments are not well known. Recent studies reported that mRNA fragments were wrapped into exosomes for functions in cells to cells communication (Mercer et al., 2011; Batagov and Kurochkin, 2013). In our study, the DE mRNA fragments were involved in several digestion-related pathways such as digestion and absorption of proteins and fats, thus suggesting that these degraded mRNAs have functions in digestion. Nevertheless, the specific mechanism still needs further research and remains to be explored.

Correlation analyses between hub miRNAs and intestinal microbiota revealed that miRNA expression in the intestine and intestinal microbiota were significantly associated. Inter-kingdom crosstalk was demonstrated between humans and bacteria by miRNAs contained in extracellular vehicles (EVs), when miRNAs generated by intestinal epithelial cells modulated bacterial gene expression to promote the growth of *Fusobacterium nucleatum* and *Escherichia coli*, which have been identified to drive colorectal cancer (Liu et al., 2016). Fecal miRNA expressions in inflammatory bowel disease (IBD) patients influenced the development of IBD by regulating of the growth of certain bacteria (Ji et al., 2018). Also, knockout of a specific miRNA, miR-21, in the mouse influence the intestinal microbiota to aggravate colitis (Johnston et al., 2018). Collectively, these altered miRNAs may play important roles in communication between the host and its intestinal bacteria and may function in genetic exchanges among cells (Valadi et al., 2007; Batagov and Kurochkin, 2013). In our study, miR-6622-3p, a DE miRNA had lower expression levels in HWS and was negatively correlated with *Alistipes putredinis*, which is energy metabolism related and had a higher abundance in HWS than LWS. Moreover, miR-6622-3p is also negatively correlated with microbial function, carbohydrate digestion and absorption, suggesting miR-6622-3p has essential roles in interactions between the host and its intestinal microbes, in the context of body weight phenotypes during evolution. miR-6622-3p also interacted with *Helicobacter pullorum*, which belongs to the family Helicobacteraceae whose heritability was moderate in the Virginia body weight lines (Meng et al., 2014), suggesting modification of its relationship was influenced by long-term selection for body weight.

In conclusion, sncRNA-mediated host regulation of intestinal microbiota can be an effective strategy for ensuring that hosts and intestinal bacteria are structured in symbiotic, co-evolutionary, and co-metabolic partnerships to build a holobiont for better adaptability to a dynamic and variable environment. Our research offers additional clues for the holobiont theory that sncRNAs have an essential role between the host and its intestinal microbes to establish a holobiont.

## Data Availability Statement

The sequencing raw data of small non-coding RNA and metagenome analyzed during this study are available in the Sequence Read Archive (https://www.ncbi.nlm.nih.gov/sra) with the accession code PRJNA601115.

## Ethics Statement

The animal study was reviewed and approved by the Institutional Animal Care and Use Committee at Virginia Tech. Written informed consent was obtained from the owners for the participation of their animals in this study.

## Author Contributions

HZ, YZ, PS, and HM were the principal investigators and project managers in this research. PS and CFH produced the chicken lines. LY, YZ, JD, CQ, CH, CFH, PS, and HM conducted the sample collection, small RNA sequencing, and metagenome sequencing. HZ, LY, JD, KX, HL, JL, YmZ, and WZ performed the correlated analysis. HZ, CHa, and KC did the integrated analysis of sequencing data. HZ and YZ submitted the sequence data to MG-RAST and NCBI. HZ, YZ, CFH, PS, and HM wrote and edited the manuscript. HZ, YZ, and HM did the final editing of the text, tables, and figures. All authors contributed to the article and approved the submitted version.

## Conflict of Interest

The authors declare that the research was conducted in the absence of any commercial or financial relationships that could be construed as a potential conflict of interest.

## Publisher’s Note

All claims expressed in this article are solely those of the authors and do not necessarily represent those of their affiliated organizations, or those of the publisher, the editors and the reviewers. Any product that may be evaluated in this article, or claim that may be made by its manufacturer, is not guaranteed or endorsed by the publisher.

## Footnotes

1
http://huttenhower.sph.harvard.edu/kneaddata

2
https://github.com/hyattpd/Prodigal

3
https://www.ncbi.nlm.nih.gov/grc/chicken

4
https://david.ncifcrf.gov/

## Supplementary Material

Supplementary Table 1 Sequencing read information.

Supplementary Table 2 DE microbes at each level.

Supplementary Table 3 Differential KEGG pathways and ECs between HWS and LWS.

Supplementary Table 4 The proportion of each type of small RNA.

Supplementary Table 5 Expression profiles of each type of small RNA.

Supplementary Table 6 Differential profiling of small RNAs of HWS and LWS.

Supplementary Table 7 DE miRNA and mRNA fragment functions.

Supplementary Table 8 Modules and hub genes.

Supplementary Table 9 Hub gene network.
