# Supplementary material for: Dynamics of Small Non-coding RNA Profiles and the Intestinal Microbiome of High and Low Weight Chickens
Source: Front Microbiol. 2022 Jun 30;13:916280. doi: 10.3389/fmicb.2022.916280 (PMC9279615; doi:10.3389/fmicb.2022.916280)
Supplement: Supplementary file 10 [file Data_Sheet_10.docx]

**Dynamics of small non-coding RNA profiles and the intestinal microbiome of high and low weight chickens**

Hao Zhou^1^, Lingyu Yang^1^, Jinmei Ding^1^, Ke Xu^1^, Jiajia Liu^1^, Wenqi Zhu^1^, Chuan He^1^, Jianshen Zhu^1^, Fisayo T. Akinyemi^1^, Chengxiao Han^1^, Chao Qin^1^, Huaixi Luo^1^, Kangchun Chen^1^, Yuming Zhen^1^, Christa F. Honaker^2^, Yan Zhang^3,*^, Paul B. Siegel^2,*^, He Meng^1,*^

^1^ Shanghai Collaborative Innovation Center of Agri-Seeds / School of Agriculture and Biology, Shanghai Jiao Tong University, Shanghai, 200240, P. R. China

^2^ Department of Animal and Poultry Sciences, Virginia Tech, Blacksburg, Virginia, 24061, USA

^3^ Carilion Clinic, Roanoke, Virginia, 24011, USA

^*^ Correspondence:

Yan Zhang: yzhang1@carilionclinic.org

Paul Siegel: pbsiegel@vt.edu

He Meng: menghe@sjtu.edu.cn

**Additional results**

**Gene catalogue of gut microbes**

We sequenced the metagenome of 20 individuals using Illumina platform and obtained 139.6 gigabases (Gb) raw data, with an average of 6.98 Gb for each sample. After quality control, the average was 6.76 Gb data for each sample, totaling 135.1 Gb of high-quality data which were free of adaptor and chicken DNA contaminants (Table S1). Based on these data, we constructed a gene catalogue from 10 high weight selected chickens (HWS) and 10 low weight selected chickens (LWS) using the methodology developed by MetaHIT. The difference in coding the sequences of 369,737±44,622 in HWS, and 427,532±67,503 in LWS was validated by multiple t’ test (P=0.046). After clustering all the coding sequences, the set of 222,7868 non-redundant CDS of the catalogue was established.

**Functional alterations of HWS and LWS microbiome**

Functional analyses were performed by eggnog-mapper based on the eggNOG database (v4.5.1). After comparison of the two groups, 26 KEGG pathways were differently enriched (q<0.01, Welch’s t-test, multiple test correction by Benjamini Hochberg FDR) (Table S3a). The enrichment of most pathways was associated with energy metabolism-related pathways. Carbon metabolism (ko01200), Carbon fixation pathways in prokaryotes (ko00720), Pyruvate metabolism(ko00620), Fatty acid degradation(ko00071), Carbohydrate digestion and absorption(ko04973), Lipoic acid metabolism(ko00785) were enriched in HWS, while pathways of Phospholipase D signaling pathway(ko04072), Phosphatidylinositol signaling system (ko04070), and Chloroalkane and chloroalkene degradation (ko00625) were more abundant in LWS. These pathways were related to the pathological state of the gut. Considering the KEGG enzymes in the gut may be responsible for different functional microbiota between two groups, we further identified 49 remarkably different abundant enzymes between them (Table S3b). The results showed the enrichment of 2-amino-3-ketobutyrate CoA ligase (2.3.1.29), xylose isomerase (5.3.1.5), and mannosylglucose phosphorylase (2.4.1.281), which are associated with energy metabolism, in HWS, but 4-hydroxybutanoyl-CoA dehydratase (4.2.1.120), UDP-N-acetylglucosamine 4,6-dehydratase (4.2.1.135), and mannitol-1-phosphate 5-dehydrogenase (1.1.1.17) were preferentially abundant in LWS. These results showed that the pathways involved in generating precursor metabolites and energy were significantly different between the two groups, as were the pathways involved in amino acid biosynthesis and nucleotide metabolism.
